# PB.52: Incidence and clinical significance of focal breast uptake at 18-fluorine fluorodeoxyglucose PET-CT in patients without known breast cancer

**DOI:** 10.1186/bcr3552

**Published:** 2013-11-08

**Authors:** P Arce-Calisaya, J Simpson, N Sharma, A Scarsbrook

**Affiliations:** 1 Saint James's University Hospital, Leeds, UK

## Introduction

18-Fluorine fluorodeoxyglucose (FDG) PET-CT is a sensitive technique and hypermetabolic foci unrelated to the primary malignancy are not infrequently encountered. The aim of this study was to evaluate the clinical significance of unexpected focal FDG uptake within the breast in patients undergoing PET-CT for assessment of other malignancies.

## Methods

Consecutive adult patients undergoing FDG PET-CT for assessment of a nonbreast primary cancer between February 2009 and October 2012 were retrospectively reviewed. The incidence of focal FDG uptake within the breast was determined. PET parameters including maximum standardised uptake value (SUVmax), metabolic tumour volume, and total lesion glycolysis were recorded for each patient. The presence and patterns of morphologic changes on CT were assessed. Aetiology and clinical significance were confirmed histologically or by imaging and clinical follow-up.

## Results

A total 23 of 8,972 patients (0.25%) had unexpected focal FDG uptake in the breast. Twenty patients (86%) underwent biopsy. Malignancy was confirmed in 17 patients, five patients (22%) had disseminated disease from their primary cancer and 12 patients (52%) had an unsuspected synchronous breast cancer. Eight patients (67%) with newly diagnosed breast cancer were outside the screening programme (≤47 ≥73 years). Only five patients (42%) had a breast lesion with SUVmax >4. No PET parameters reliably distinguished benign from malignant pathology.

## Conclusion

Incidental focal FDG uptake in the breast is rare but requires further evaluation as approximately 50% of cases may reflect unsuspected breast malignancy.

